# Loss of *runx1* function results in B cell immunodeficiency but not T cell in adult zebrafish

**DOI:** 10.1098/rsob.180043

**Published:** 2018-07-25

**Authors:** Yali Chi, Zhibin Huang, Qi Chen, Xiaojie Xiong, Kemin Chen, Jin Xu, Yiyue Zhang, Wenqing Zhang

**Affiliations:** 1 Key Laboratory of Zebrafish Modeling and Drug Screening for Human Diseases of Guangdong Higher Education Institutes, Department of Developmental Biology, School of Basic Medical Sciences, Southern Medical University, Guangzhou 510515, People's Republic of China; 2 Division of Cell, Developmental and Integrative Biology, School of Medicine, South China University of Technology, Guangzhou 510006, People's Republic of China; 3 Department of Obstetrics and Gynecology, Nanfang Hospital, Southern Medical University, Guangzhou 510515, People's Republic of China

**Keywords:** *Runx1* mutation, lymphocyte, immunodeficiency, zebrafish

## Abstract

Transcription factor RUNX1 holds an integral role in multiple-lineage haematopoiesis and is implicated as a cofactor in V(D)J rearrangements during lymphocyte development. *Runx1* deficiencies resulted in immaturity and reduction of lymphocytes in mice. In this study, we found that *runx1^W84X/W84X^* mutation led to the reduction and disordering of B cells, as well as the failure of V(D)J rearrangements in B cells but not T cells, resulting in antibody-inadequate-mediated immunodeficiency in adult zebrafish. By contrast, T cell development was not affected. The decreased number of B cells mainly results from excessive apoptosis in immature B cells. Disrupted B cell development results in *runx1^W84X/W84X^* mutants displaying a similar phenotype to common variable immunodeficiency—a primary immunodeficiency disease primarily characterized by frequent susceptibility to infection and deficient immune response, with marked reduction of antibody production of IgG, IgA and/or IgM. Our studies demonstrated an evolutionarily conserved function of *runx1* in maturation and differentiation of B cells in adult zebrafish, which will serve as a valuable model for the study of immune deficiency diseases and their treatments.

## 1. Introduction

Multipotent haematopoietic stem cells germinate lymphoid-restricted progenitors that differentiate into subsets of B and T cells. Lymphocytes play a vital role in adaptive immunity, while B cells provide varied immunoglobulin antibodies which perform the humoral immune response [1]. The matured B cells can differentiate into plasmocytes and memory cells to eliminate pathogens and maintain the health of individuals via the immune response. Therefore, the dysregulation of the production and functionality of B cells often results in a variety of human diseases, including leukaemia [2], common variable immunodeficiency (CVID) [3] and X-linked agammaglobulinaemia [4]. CVID refers to a heterogeneous collection of primary immunodeficiency diseases, primarily characterized by frequent susceptibility to infection and deficient immune response, concomitant with a marked reduction in antibody production of IgG, IgA and/or IgM. Defective lymphocyte development, especially of B cells, is the major pathophysiological cause of CVID [5].

Mammalian B cell development is a sequential process, which can be divided into seven stages: pre-pro-B cell, pro-B cell, pre-B cell, immature B cell, mature B cell, activated B cell and plasma B cell [6,7]. V(D)J rearrangements of B cells are responsible for producing the Ig heavy chains during the B cell maturation. Complex pathways and multiple genes drive B lymphopoiesis, including B cell maturation, commitment, specification and differentiation. Mouse and cell line studies have shown that *Pax5* (B cell-specific activator protein, BSAP) has concerted action with a set of genes such as *Runx1*, *Blk*, *E2a*, *Ikaros* and other B-lineage-specific transcription factors to establish the B cells development network [6,8–10]. Coordination failure between different signalling molecules and regulators would result in defects in B lymphopoiesis. RUNX1 is a critical member of the RUNX (runt-related) family harbouring highly conserved DNA binding and protein–protein interaction domains as heterodimeric transcription factors in vertebrate [11–14]. Mutations in *Runx1* are known to be strongly and frequently associated with haematological malignancies [15], where RUNX1 serves as a key regulator in the initiation and maintaining a steady state of haematopoietic stem cell [16–18], the emergence of thrombocytes [19] and growth of lymphocytes [20–22]. A recent survey study concluded that of 128 patients with acute lymphoblastic leukaemia, approximately 18.3% patients with T cell acute lymphoblastic leukaemia and 3.8% patients with B cell acute lymphoblastic leukaemia carried a *RUNX1* mutation [23]. Several genes have been shown to be the downstream targets of *Runx1*, including *Ebf1*, *Ly6d*, *Spib* and *Ikzf3* [20,21]. However, the mechanism and signalling pathway of RUNX1 in modulating lymphocyte development remain incompletely elucidated.

Haematopoietic processes of zebrafish are evolutionarily similar to mammalian processes, including lymphopoiesis [24]. Zebrafish has been an excellent vertebrate genetic and developmental system for disease analysis, contributing a valuable increase in the understanding of haematopoiesis and the immune system [25,26]. In zebrafish, the thymus is generally the first to develop as a lymphoid organ accumulating T cells [27], which is initiated by expression of *rag1/2*. B cells emerge from 21 days post-fertilization (dpf) in the pronephros and kidney marrow [28], in which large antibody repertoires exist [29]. The conserved haematopoietic programme of zebrafish has served as a versatile model organism to demonstrate the events in vertebrate lymphoid ontogeny.

The tightly regulated network of *runx1* activation has been studied extensively in humans and mice, but not in adult zebrafish. We have established a CVID model by utilizing zebrafish *runx1^W84X/W84X^* mutants, which mimic the haematopoietic and immunodeficiency of B cells. Using these mutants, we address how *runx1* regulates B cell growth and the mechanism of CVID. Our results indicated a dramatic decrease of B cell numbers, ineffective immune response and aborted V(D)J rearrangements of B cells in *runx1^W84X/W84X^* mutants, demonstrating a conserved role of *runx1* in B cell development. This model can be used for exploring potential therapies for CVID.

## 2. Results

### 2.1. Abnormal development of lymphocytes in *runx1^W84X/W84X^* mutants

**Figure 1.**
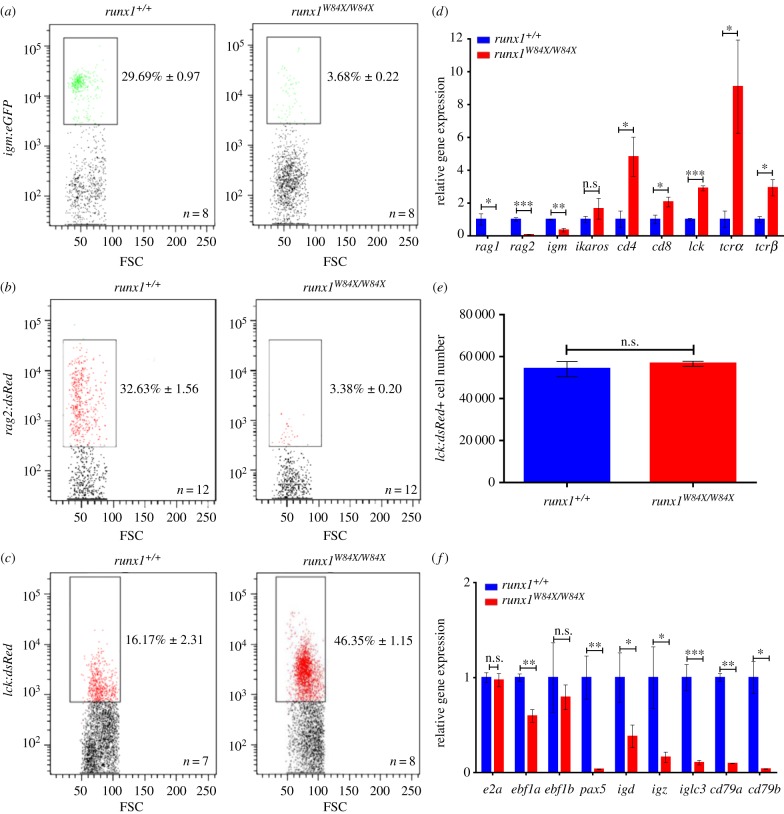
Aberrant developmental characterization of lymphocytes in adult *runx1^W84X/W84X^* mutants. (*a–c*) FACS analysis of B cell makers *igm* (*a*) and *rag2* (*b*), T cell maker *lck* (*c*) in kidney marrow of *runx1^+/+^* and *runx1^W84X/W84X^* mutants. Black boxes outline the captured positive lymphocytes with fluorescence in lymphocytic populations of kidney marrow; the percentage data represents the mean ± s.e.m. (*d*) Relative expression of B cell makers and T cell makers in *runx1^+/+^* (blue bars, *n* = 54) and *runx1^W84X/W84X^* mutants (red bars, *n* = 54) were examined by Q-RT-PCR. (*e*) Absolute numbers of T cells in *runx1^+/+^* (blue bar, *n* = 3)and *runx1^W84X/W84X^* mutants (red bar, *n* = 3). (*f*) Relative expression of genes important at multiple stages of B cells development. Each experiment was performed in duplicate. Unpaired Student's *t*-test; ns, no significance; *p < 0.05; **p < 0.01; ***p < 0.001.

*Runx1* is essential for survival and for the continued development of B cells and T cells in mice [20,21,30,31]. To gain insights into the roles of *runx1* in adult zebrafish lymphocyte development, we used *runx1^W84X/W84X^* mutants that produce truncated proteins and lack *runx1* function [32,33]. As in other teleosts, adult zebrafish maintain multi-lineage haematopoiesis in the kidney marrow, an organ that is equivalent to the mammalian bone marrow, the source of B cells and T cells. We used *Tg(igm:eGFP)*, *Tg(rag2:dsRed)* and *Tg(lck:dsRed)* transgenic lines; these closely recapitulate mammalian B cell [34,35] and T cell ontogeny, respectively. FACs analysis demonstrated that *runx1^W84X/W84X^* mutants showed sharply reduced percentages of *igm:eGFP^+^* and *rag2:dsRed^+^* B cells but expanded *lck:dsRed^+^* T cells compared with *runx1^+/+^* (figure 1*a–c*). We then examined the expression of B-cell- and T-cell-related genes in lymphocytic populations of kidney marrow from adult *runx1^+/+^* and *runx1^W84X/W84X^* mutants using Q-RT-PCR [34]. Specific primers were designed as seen in table 1. As expected, we found decreased B cell genes and increased T cell genes (figure 1*d*), indicating that T cell number may not be affected or may even increase. To more accurately discern whether *runx1* would affect T cell development, we measured T cell number in the kidney (figure 1*e*). The absolute T cell number in *runx1^W84X/W84X^* mutants is comparable to that of *runx1^+/+^*. Therefore, the increase in T cell percentage is likely to be the mathematical consequence of the reduction in B cell percentage, rather than the increase of T cell quantity.

**Table 1.** Primers used in Q-RT-PCR.

gene	FP	RP
*ef-1α*	TACTTCTCAGGCTGACTGTG	ATCTTCTTGATGTATGCGCT
*rag1*	AATGATGCAAGGCAGAGGA	CAATGATGCCCACATCCC
*rag2*	TGAGACTCAGAAGCGCATGG	ACCAAGTACGACTGTGGCTG
*igm*	GTTTCCTCAGCTCAACCA	AGTATAATCTCCTTCCTTCCC
*ikaros*	AGAAGG GTAACCTGCTCCGACAC	GGGCTT TCCAACCGAATGAGT
*cd4*	GTGGTCTTCATCTTGCTTGT	AATCCCTTTGGCTGTTTGTT
*cd8*	AAGAGCATAGCACCGTAG	GACTTCCGTCTGCTTTGCG
*lck*	ACGTAAACATGGGGAACTG	TCTTCTCCCCTTTCTCAAAC
*tcrα*	GAAGCCGAATATTTACCAAGTG	AACAAACGCCTGTCTCCT
*tcrβ*	AAATCAACAAACAAATTCACCTG	TATGCCAGCTTCATCCACTG
*e2a*	AATGTGCAAGAAGGACTTCCAGATC	CATGATGCCTTCGCTGGAGCTGAT
*ebf1α*	TTTACAGCAACGGCATCAGAACAG	GGTTACATTTGAGGAAGAATTTCAGG
*ebf1β*	ATCATATTTGGAGCATGCTGCACCT	CTTATAGGAGAGTGTGACCTCTACC
*pax5*	CTGATTACAAACGCCAAAAC	CTAAATTATGCGCAGAACG
*igd*	GACACATTAGCCCATCAGCA	CTGGAGAGCAGCAAAAGGAT
*igz*	AAAGCAACGATACCAAAGTG	AACAGCTTGCAAGACAATTC
*iglc3*	AAGGAACTAAACCCATTGTGACGGA	TCGCTGCATTCAGATTTCCTGATG
*cd79a*	TCAAGAATACTCCCGCCATC	GGCTTCTCCAGCTGAATGTC
*cd79b*	GCTCACTTACGAATGACCAGAGAATAAC	GTCCTCATACACATCTCCACCAACC
*distal primer*	ACTAGATGACAATGTTGCGCTGGCAAC	CAGTTGGGGGTAATTATGACTAACAAAAGTGCT
*proximal primer*	GACAGCTAATGGTAGTTCGGCTTACTTATG	CTTGTGGAGACAGCTCCCTCGCTGTTC

Additionally, B cells from another immune organ, spleen, were reduced in *runx1^W84X/W84X^* mutants (electronic supplementary material, figure S1A,B). To determine the precise developmental stages during which B cell differentiation is blocked, we analysed the expression of B-cell-specific genes such as *e2a*, *ebf1α*, *ebf1b*, *pax5*, *igd*, *igz, iglc3*, *cd79a* and *cd79b* which are important at multiple stages of B cell development [36–38] (figure 1*f*). These results indicated that B cells had been deficient from an early stage of B cell development in *runx1^W84X/W84X^* mutants, similar to the *Runx1* knock-out mouse [20].

### 2.2. B cells but not T cells are dysfunctional in *runx1^W84X/W84X^* mutants: establishment of common variable immunodeficiency model

**Figure 2.**
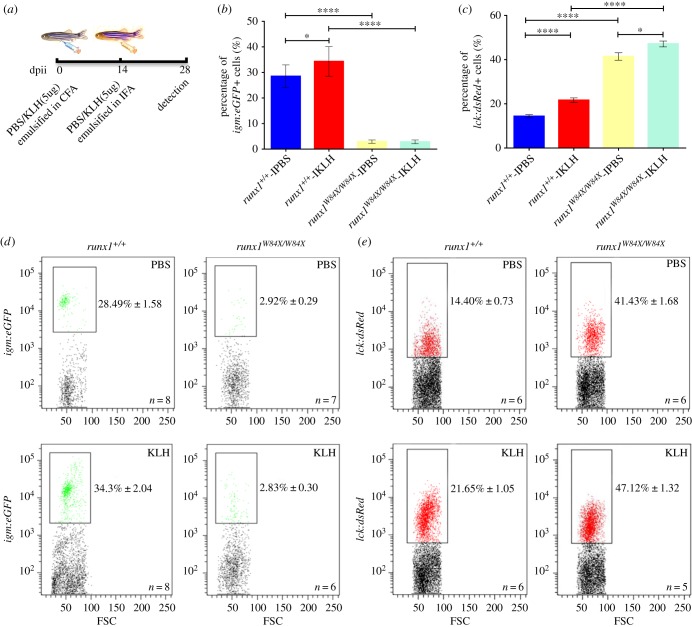
Deficient immune function of B cells but not T cells in adult *runx1^+/+^* and *runx1^W84X/W84X^* mutants kidney marrow. (*a*) Scheme of immune responses procedure showing dose, assigned groups and process involved in intraperitoneal injection of KLH. Percentage of *igm:eGFP^+^* and *lck:dsRed^+^* cells in lymphocytes of kidney marrow were detected at 28dpii. dpii, day post intraperitoneal injection; PBS, phosphate buffer solution; KLH, keyhole limpet haemocyanin; CFA, complete Freund's adjuvant; IFA, incomplete Freund's adjuvant. (*b–e*) Quantified percentage and FACS analysis of *igm:eGFP^+^* cells (*b,d*) and *lck:dsRed^+^* cells (*c,e*) in lymphocytes of kidney marrow from assigned groups at 28dpii. Each experiment was performed in duplicate. Blue bars indicate the *runx1^+/+^*-IPBS, red bars indicate the *runx1^+/+^-*IKLH, yellow bars indicate the *runx1^W84X/W84X^*-IPBS, light blue bars indicate the *runx1^W84X/W84X^*-IKLH; black boxes outline the captured positive lymphocytes with fluorescence in lymphocytic population of kidney marrow; ANOVA, Dunnett T3; *p < 0.05; **p < 0.01; ***p < 0.001; ****p < 0.0001.

We observed that although *runx1^W84X/W84X^* mutants were similar in size to *runx1^+/+^*, 35 of 687 (about 5.09%) *runx1^W84X/W84X^* mutants appeared frail and ill, suffering recurrent infection and displaying anomalies in sustained swimming and morphology (electronic supplementary material, figure S2A). By recording the gross survival rate of *runx1^+/+^* and *runx1^W84X/W84X^* mutants every day, we found a much lower survival rate of *runx1^W84X/W84X^* mutants compared with *runx1^+/+^* (electronic supplementary material, figure S2B). The dysplasia of B cells and T cells prompted us to test the immune function of *runx1^W84X/W84X^* mutants by measuring the immune response [35,39]. To this end, phosphate buffer solution (PBS) as the control and KLH emulsified in complete Freund's adjuvant were used as antigen to immune *Tg(igm:eGFP);runx1^+/+^* and *Tg(igm:eGFP);runx1^W84X/W84X^* mutants, *Tg(lck:dsRed);runx^+/+^* and *Tg(lck:dsRed);runx1^W84X/W84X^* mutants. Two weeks later, PBS and KLH emulsified in incomplete Freund's adjuvant (IFA) boosted the immune fish (figure 2*a*). After four weeks, the percentage of *igm:eGFP^+^* B cells in lymphocytes were increased in *Tg(igm:eGFP);runx1^+/+^*, but not in *Tg(igm:eGFP);runx1^W84X/W84X^* mutants (figure 2*b,d*). The data indicated that the residual B cells in *runx1^W84X/W84X^* mutants lacked immune response to antigen stimulation. In contrast, compared with *Tg(lck:dsRed);runx^+/+^*, the immune response of *lck:dsRed^+^* T cells in *Tg(lck:dsRed);runx1^W84X/W84X^* mutants was normal (figure 2*c,e*). These data provided the conclusion that loss of *runx1* function in zebrafish resulted in dysfunctional development of B cells but not that of T cells, a phenotype resembling CVID with reduced B cells, defects in B cell development, and impaired secretion of immunoglobulin in humans [40].

### 2.3. Apoptosis of B cells is increased in *runx1^W84X/W84X^* mutants

**Figure 3.**
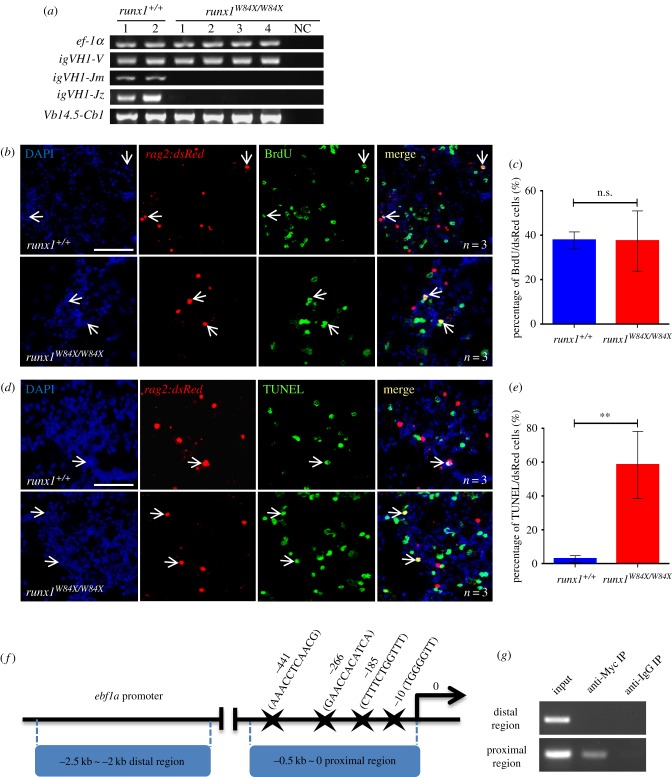
Deficiency of B cells development in *runx1^W84X/W84X^* mutants. (*a*) V(D)J rearrangements of *igm*, *igz* and V(DJ)C rearrangement of *tcrβ* analysis in kidney marrow from *runx1^+/+^* (*n* = 2) and *runx1^W84X/W84X^* mutants (*n* = 4) by semi-nested PCR. The *ef-1α* and *igVH1-V* PCR were used as positive control. NC, negative control. (*b–c*) Cell proliferation assay of B cells in kidney marrow of *runx1^+/+^* and *runx1^W84X/W84X^* mutants. (*b*) Triple staining of DAPI, *rag2:dsRed* and BrdU. Blue: DAPI, red: dsRed, green: BrdU; arrows indicate DAPI staining, *rag2:dsRed* staining, BrdU staining and triple co-staining cells. (*c*) Comparison of percentage of *rag2:dsRed^+^* cells for BrdU co-staining cells between *runx1^+/+^* (blue bar, *n* = 3) and *runx1^W84X/W84X^* mutants (red bar, *n* = 3). Unpaired Student's *t*-test; *p <* 0.05; ns, no significance; mean ± s.e.m. (*d–e*) Cell apoptosis assay of B cells in kidney marrow of *runx1^+/+^* and *runx1^W84X/W84X^* mutants. (*d*) Triple staining of DAPI, *rag2:dsRed* and TUNEL. Blue: DAPI, red: dsRed, green: TUNEL; arrows indicate DAPI staining, *rag2:dsRed* staining, TUNEL staining, and triple co-staining cells. (*e*) Comparison of percentage of *rag2:dsRed^+^* cells for TUNEL between *runx1^+/+^* (blue bar, *n* = 3) and *runx1^W84X/W84X^* mutants (red bar, *n* = 3). (*f*) Schematic diagram of the 2.5 kb *ebf1a* promoter region. The transcription initiation site is designated as 0. Putative Runx1 consensus sites (marked by stars) are shown. (*g*) Semi-quantitative PCR analysis of the enrichment of the −0.5 ∼ 0 kb proximal region (ii) and the −2.5 ∼ −2 kb distal region (i). The left lanes were input DNA control. Each experiment was performed in duplicate. Unpaired Student's *t*-test; mean ± s.e.m; **p < 0.01; scales bars, 100 µm.

It is known that lymphocytic V, D and J gene segment rearrangements are the initial essential events that occur in the maturation of B cells and T cells. Adult *runx1^W84X/W84X^* mutants have abnormal development of B cells and/or T cells. We intended to detect V(D)J rearrangements of B cells and T cells in kidney from *runx1^+/+^* and *runx1^W84X/W84X^* mutants by semi-nested PCR assays. The expression of Ig heavy chain isotypes *igm* and *igz*, in addition to T cell receptor *tcrβ* rearrangements, were checked by the primers listed in table 2 [41–43]. *Igm*, *igz* and *tcrβ* were robustly amplified in both *runx1^+/+^* and *runx1^W84X/W84X^* mutants. *runx1^W84X/W84X^* mutants had nearly undetectable *igm* and *igz* rearrangement expression but had normal *tcrβ* transcriptional rearrangement expression in the kidney (figure 3*a*). These results imply that, similar to the *Runx1*-null mutation in mice, B cell rearrangements were interrupted in *runx1^W84X/W84X^* mutants. This suggests a functional requirement of *runx1* in the maturation and/or maintenance of B cells in zebrafish. Moreover, we found that in zebrafish, *runx1*'s role is indispensable in the development of B cell but not T cells.

**Table 2.** Primers used in rearrangement assays.

gene	FP	RP
*ef-1α*	TATCTCCAAGAACGGACAGAC	GCAAACTTGCAGGCGATGTG
*igVH1-V* (first round)	GATGGACGTGTTACAATTTGG	CGTGCACAGTAATAAACAGCT
*igVH1-V* (second round)	CCTCCTCAGACTCTGTGGTGA	CGTGCACAGTAATAAACAGCT
*igVH1-Jm* (first round)	GATGGACGTGTTACAATTTGG	GTTCCYTTHCCCCAGTAGTCAAA
*igVH1-Jm*(second round)	CCTCCTCAGACTCTGTGGTGA	GTTCCYTTHCCCCAGTAGTCAAA
*igVH1-Jz* (first round)	GATGGACGTGTTACAATTTGG	AAGGTCTATTACTAACAGATCAC
*igVH1-Jz*(second round)	CCTCCTCAGACTCTGTGGTGA	AAGGTCTATTACTAACAGATCAC
*Vb14.5-Cb1*(first round)	GAATCCAATGTGACGTTAACATGC	AAGATGACAAGGCCATACAGTC
*Vb14.5-Cb1*(second round)	CATGATCATAAGGACCACTACAG	GTCCGCTCTTAGCAATGGTC

In theory, reduced B cells in kidney from *runx1^W84X/W84X^* mutants could be due to any one or more of the three following underlying cellular abnormalities: (1) impaired proliferation of existing B cells; (2) reduced de novo B cells; (3) enhanced apoptosis of B cells. In the light of the essential role of *runx1* in proliferation and cell apoptosis, we next monitored the proliferation and cell apoptosis of B cells via BrdU incorporation assay and TUNEL (terminal deoxynucleotidyl transferase dUTP nick end labelling) assay [44] respectively to survey which cellular mechanisms mediated the reduced B cells in *runx1^W84X/W84X^* mutants. Quantification of the DAPI/*rag2:dsRed*/BrdU triple positive cells population showed the B cell proliferation was similar between *runx1^+/+^* and *runx1^W84X/W84X^* mutants (figure 3*b,c*). However, measurement of the DAPI/*rag2:dsRed*/TUNEL triple positive cell population showed a significantly increased ratio in *runx1^W84X/W84X^* mutants compared with *runx1^+/+^*, suggesting that B lymphocyte apoptosis was increased (figure 3*d,e*).

It was reported that *Runx1* controls B lymphopoiesis by regulating *Ebf1* [20], and loss of *Ebf1* results in increased apoptosis in pro-B cells [45]. Many EBF1 target genes, for example, components of the pre-BCR and BCR, *Cd79a*, *Cd79b*, B lymphoid kinase, *Vpreb1*, *Igll1* and *Cd19* genes, are required for B cell survival [46]. In this study, we found that *runx1^W84X/W84X^* mutation led to the reduction of *ebf1*, *pax5*, *rag1/2*, *cd79a* and *cd79b*, which are necessary for B cell survival (figure 1*d,f*). In addition, we detected the binding of Runx1 to the zebrafish *ebf1a* promoter. Four putative Runx1 consensus sites identified within −0.5 ∼ 0 kb proximal region using JASPAR online software, with their positions (marked by stars) relative to the transcription initiation site, are shown (figure 3*f*). Chromatin immunoprecipitation (ChIP) showed that Myc-tagged Runx1 binds to the −0.5 ∼ 0 kb proximal but not distal promoter region (−2.5 ∼ −2 kb) of the *ebf1a* promoter (figure 3*g*). These Runx-binding motifs may be essential for gene expression, suggesting that Runx1 may directly control the transcription of *ebf1a*. Therefore, *runx1* is likely to promote B cell development by regulating key factors such as *ebf1a*, which in turn regulates *pax5*, its known downstream target, to control the development of B cells.

### 2.4. *Runx1* regulates B cells development in a cell-autonomous manner in zebrafish

**Figure 4.**
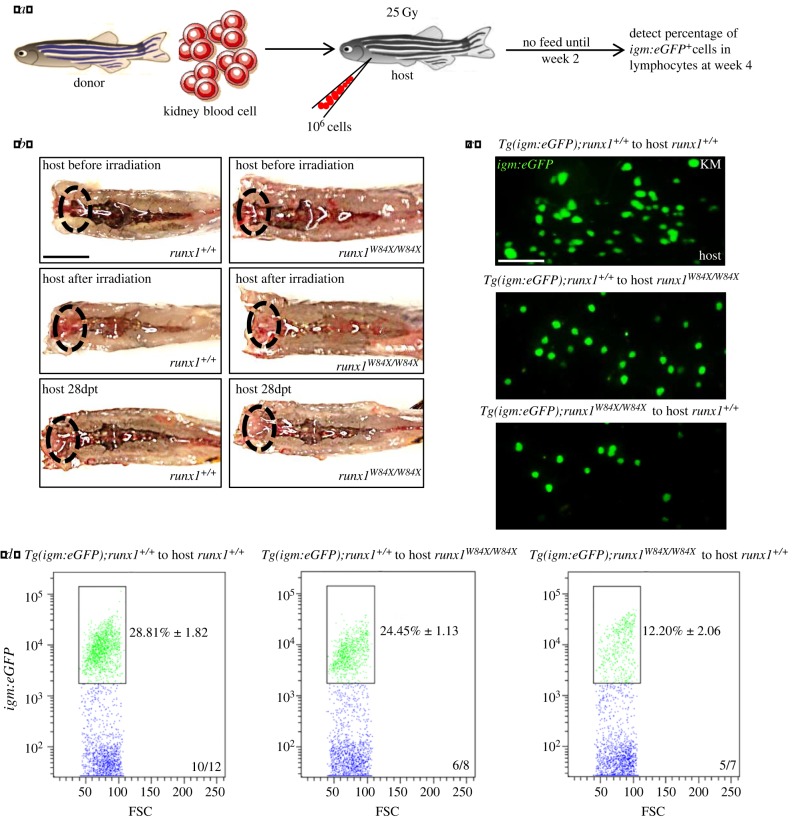
Cell-autonomous regulation of *runx1* in B cells development. (*a*) Schematic outline showing procedure of transplantation. (*b*) Macroscopic representation of kidney marrow of donors (top), donors after irradiation (middle) and hosts reconstituted with donors kidney morrow (below, 4 weeks after transplantation). Ovals indicate the locations of the head kidneys; dpt, day post transplantation; scales bars, 5 mm. (*c*) Fluorescent representation of *igm:eGFP^+^* from reconstituted kidney morrow of hosts (4 weeks after transplantation). Scales bars, 100 µm. (*d*) Percentage of *igm:eGFP^+^* in lymphocytes from reconstituted hosts kidney marrow were calculated by FACS analysis. Each experiment was performed in duplicate. Black boxes outline the captured *igm:eGFP^+^* cells with fluorescence in lymphocytes region of kidney marrow.

The importance of the expression of *runx1* in haematopoiesis raised the question of whether *runx1* is required cell-autonomously or non-cell-autonomously for the development of B cells in *runx1^W84X/W84X^* mutant zebrafish. To determine between the two possibilities, we conducted reciprocal kidney transplantation experiments between *runx1^+/+^* and *runx1^W84X/W84X^* mutants [47–49] (figure 4*a*). We transplanted whole kidney blood cells from *Tg(igm:eGFP);runx1^+/+^* donor to *runx1^+/+^* host, *Tg(igm:eGFP);runx1^+/+^* donor to *runx1^W84X/W84X^* mutant host and *Tg(igm:eGFP);runx1^W84X/W84X^* mutant donor to *runx1^+/+^* host. We found that in *Tg(igm:eGFP);runx1^+/+^* to *runx1^+/+^* group, 10 of 12 hosts have *runx1^+/+^*-phenotyped B cell reconstitution (figure 4*b–d*); in *Tg(igm:eGFP);runx1^+/+^* to *runx1^W84X/W84X^* group, 6 of 8 hosts have *runx1^+/+^*-phenotyped B cell reconstitution; and in *Tg(igm:eGFP);runx1^W84X/W84X^* mutants to *runx1^+/+^* group, 5 of 7 hosts *runx1^+/+^* displayed B cell phenotype identical to *runx1^W84X/W84X^* mutants (figure 4*b–d*). In brief, these data strongly argued that *runx1* regulates development of B cells through a cell-autonomous manner in zebrafish.

## 3. Discussion

In our study, we confirmed that the necessity of *runx1* in B lymphopoiesis of adult zebrafish is conserved. We established the CVID model in adult zebrafish with *runx1^W84X/W84X^* mutation, which demonstrates a phenotype consistent with that of adult *Runx1*-null mice. A decreased ratio of B cells with an increased ratio of T cells was observed in adult zebrafish kidney with *runx1* absence. From the reduced *igm:eGFP^+^* and *rag2:dsRed^+^* B cells, we recapitulated that the mature and immature B cells were both decreased in *runx1^W84X/W84X^* mutants kidney and spleen. This result could be explained by the previous research that *rag2*-positive cells are likely to be restricted to immature B cells in the kidney [34]. Furthermore, we found that the absolute T cell number in *runx1^W84X/W84X^* mutants is comparable to that of *runx1^+/+^*. Nevertheless, we were not surprised to discover the increased percentage of T cells in *runx1^W84X/W84X^* mutants. It is supposed that the ratio of T cells may be relatively increased rather than absolutely increased, as it is accompanied by a reduction of B cells.

In mammals, *Runx1* is induced to express in early B cell development and maintained at the subsequent various stage of B cells [50–52]. The essential regulators *E2a*, *Ebf1* and *Pax5* are tightly associated with maturation of B cells in both mouse and human [35,53]. Our studies showed a marked decrease in the expression of *pax5* and *ebf1α* in *runx1^W84X/W84X^* mutants, consistent with previous observations in mice. To our knowledge, *runx1* could cooperate with *pax5* in activation of the *ebf1α* gene promoter in mastering the B cells development [20]. We also identified the conserved *runx1*-*ebf1α* axis regulation on the B cells development in zebrafish. We confirmed the Runx-binding motifs on *ebf1a* promoter, which suggested that Runx1 may directly control the transcription of *ebf1a* in zebrafish. Therefore, in our zebrafish model, *runx1* is likely to promote B cell development by regulating key factors such as *ebf1a*, which in turn regulates *pax5*, its known downstream target, to control the development of B cells.

Characteristics of the zebrafish immune system are comparable with that of mammals, making it a versatile model to address questions about immunity and disease. By measuring the KLH-mediated proliferation of B cells and T cells in the kidney, we found a negative immune response to antigen and crippled immunoglobulin secretion of B cells in *runx1^W84X/W84X^* mutants, suggesting that *runx1* deficiency impaired the adaptive defense system. This reminded us that the phenotypes of *runx1^W84X/W84X^* mutants were similar to CVID in human. CVID is diagnosed mainly based on decreased serum immunoglobulin levels, recurrent infection and the absence of specific antibodies against antigens [5]. CVID is intractable without clear pathogenesis. To date, it has not been reported that the lesion in *runx1* is genetically associated with CVID.

B cells with ineffective V(D)J rearrangements demonstrate immaturity, which promotes apoptosis [54]. Mechanistically, we found that absence of *runx1* increased apoptosis of B cells. This is probably due to the blocked maturation of B cells incurred by inhibited *runx1* function. In addition, by kidney transplantation, we found cell-autonomous regulation of B cells growth in adult zebrafish. This confirmed the indispensable role of *runx1* in haematopoiesis.

In conclusion, loss of *runx1* function resulted in dysfunction of B cells in adult zebrafish. We are the first to use inherited *runx1^W84X/W84X^* mutants in zebrafish with B cell deficiency to establish a powerful CVID model, which will provide chances to further explore B cell development as well as potential therapy for CVID patients.

## 4. Material and methods

### 4.1. Zebrafish strains and maintenance

Zebrafish stocks were handled and bred in standard circulating water system as described previously [55]. *runx1^W84X/W84X^* mutant zebrafish line [32,33] was kindly provided by Dr P. Paul Liu and Dr Zilong Wen. *runx1^+/+^* zebrafish were spawned to produce the *runx1^+/+^* zebrafish and homozygous *runx1^W84X/W84X^* mutants were spawned to propagate *runx1^W84X/W84X^* mutants in this study. *Tg(Cau.Ighv-ighm:eGFP)* referred to in texts as *Tg(igm:eGFP)* [35], *Tg(rag2:dsRed)zf411* referred to in texts as *Tg(rag2:dsRed)* [56], *Tg(lck:loxp-dsRed-loxp-eGFP)* referred to in texts as *Tg(lck:dsRed)* [25] *and Tg(hsp70l:MYC-runx1)hkz02t* referred to in texts as *hps70l:myc-runx1* [57] were used in this study. Genomic DNA was harvested from clipped tail fins, PCR amplification and digestion by Hae II restriction enzyme determined the genotype of *runx1^W84X/W84X^* mutants. Adult *runx1^W84X/W84X^* mutants zebrafish were mated to *Tg(igm:eGFP)*, *Tg(rag2:dsRed) and Tg(lck:dsRed)* respectively, and their embryos were harvested and raised. Then we generated *Tg(igm:eGFP);runx1^W84X/W84X^* mutants, *Tg(rag2:dsRed);runx1^W84X/W84X^* mutants and *Tg(lck:dsRed);runx1^W84X/W84X^* mutants lines*.* In all experiments, zebrafish embryos were raised in fish water consisting of 2 mg l^−1^ methylene blue in deionized water as a fungicide.

### 4.2. Flow cytometry

Haematopoietic cells obtained from adult *runx1^+/+^* and *runx1^W84X/W84X^* mutants were processed as described [58]. Haematopoietic cells isolated from kidney and spleen were resuspended using ice-cold PBS with 5% FBS, then subjected to measurement based on forward scatter and side scatter with a flow cytometer (Becton Dickinson, San Jose, CA) and results were analysed with FlowJo software (TreeStar, Ashland, OR). Lymphocytic populations in FSC^int^SSC^low^ were sorted based on forward and side scatters on flow cytometry sorter (Becton Dickinson, San Jose, CA) and was used in reverse transcription reactions. The percentage of GFP fluorescence-activated cells or dsRed fluorescence-activated cells in the lymphocytic population was analysed [59].

### 4.3. RNA extraction and quantitative RT-PCR

Total RNA from lymphocytes of adult *runx1^+/+^* and *runx1^W84X/W84X^* mutants were extracted by TRIzol Reagent (Roche, Basel, Switzerland) following the manufacturer's instructions and converted to complementary DNA (cDNA) using M-MLV Reverse Transcriptase (Promega, Madison, USA) with oligo18-dT (deoxy-thymine) primers. RNA was treated with RNase-free DNase (Thermo Fisher Scientific, Waltham, USA) before the reverse transcription reaction. Q-RT-PCR reactions were performed using LightCycler Nano System (Roche, Basel, Switzerland) with FastStart Universal SYBR Green Master (ROX) (Roche, Basel, Switzerland) with 10 pmol of each primer and each sample was tested in triplicate. The housekeeping gene, elongation factor 1-α (*ef-1α*), served as an internal control to normalize the relative fold changes using the *ΔΔ*Ct threshold method. The primers used in Q-RT-PCR are listed in table 1.

### 4.4. Analysis of V(D)J rearrangements

Kidneys were obtained from adult *runx1^+/+^* and *runx1^W84X/W84X^* mutants then subjected to genomic DNA isolation (QIAGEN, Hilden, Germany). Semi-nested PCRs were processed with published primers spanning the V(D)J-JM region of *igm* (*igVH1-Jm*), and *igz* (*igVH1-Jmz*) using the genomic DNA [41–43]. The semi-nested PCRs program was carried out as follows: first round, 1 cycle for 94°C/120 s, 30 cycles for 94°C/30 s, 61°C/30 s and 72°C/30s-90 s, 1 cycle for 72°C/180 s. After the first round of semi-nested PCR, 1 µl PCR product was diluted into 20 times as the template for the second round of semi-nest PCR. The semi-nested PCRs programme of the second round was as follows: 1 cycle for 94°C/120 s, 21 cycles for 94°C/30 s, 61°C/30 s and 72°C/30s-90 s, 1cycle for 72°C/180 s. For *tcrβ* rearrangements assay, RNA was isolated from the kidney marrow of adult *runx1^+/+^* and mutants and converted to cDNA. *tcrβ* rearrangements were PCR amplified, referred to in the figure as *Vb14.5-Cb1* using semi-nested PCRs, as previously described [42]. The first round of PCR cycle parameters were as follows: 1 cycle for 94°C/120 s, 35 cycles for 94°C/30 s, 56°C/30 s and 72°C/60 s, 1 cycle for 72°C/180 s. 1 µl PCR product was diluted into 20 times as the template for the second round of semi-nest PCR. The cycling parameters were identical to those of the first reaction, except that the steps were repeated for 25 cycles.

The *ef-1α* and *igVH1-V* PCR products used as the standard control were run in the parallel tubes. The semi-nested PCR products were analysed by 1.5% agarose gel electrophoresis and stained with ethidium bromide. The primers used in the rearrangement assays are listed in table 2.

### 4.5. Survey of immune responses

Keyhole limpet haemocyanin (KLH) (Sigma-Aldrich, St. Louis, USA) dissolved in sterile PBS fully, then emulsified in CFA (Sigma-Aldrich, St Louis, USA) and in IFA (Sigma-Aldrich, St Louis, USA) at the concentration of 5 µg µl^−1^ [35,60]. Ultrasonic emulsification on ice with 4 s, 2 s, 350 W was performed until the mixture would stay agglomerated when dropped on the ice-water. Adult *Tg(igm:eGFP);runx1^+/+^* and *Tg(igm:eGFP);runx1^W84X/W84X^* mutants with both sexes, weighing approximately 0.5–1 g, body lengths 2–3 cm were randomly prepared into four groups: the *Tg(igm:eGFP);runx1^+/+^*-PBS, the *Tg(igm:eGFP);runx1^+/+^*-KLH, the *Tg(igm:eGFP);runx1^W84X/W84X^*-PBS and the *Tg(igm:eGFP);runx1^W84X/W84X^* -KLH, to receive the detection of immune response. The four groups were immunized by intraperitoneal injection with the same volume of sterile PBS and a dose of 5 µg KLH emulsified in CFA, respectively. Followed 14 days later, the *Tg(igm:eGFP);runx1^+/+^* and *Tg(igm:eGFP);runx1^W84X/W84X^* mutants were again intraperitoneally injected with same 1 µl volume of sterile PBS and 5 µg KLH emulsified in IFA, respectively [39]. Four weeks later, the percentage and number of *igm:eGFP^+^* cells in the lymphocytic population of kidney from adult *Tg(igm:eGFP);runx1^+/+^* and *Tg (igm:eGFP);runx1^W84X/W84X^* mutants as well as *lck:dsRed^+^* cells in the lymphocytic population of kidney from adult *Tg(lck:dsRed);runx1^+/+^* and *Tg(lck:dsRed);runx1^W84X/W84X^* mutation lines were analysed to examine the function of B cells and T cells.

### 4.6. B cell proliferation and apoptosis assay

Adult *runx1^+/+^* and *runx1^W84X/W84X^* mutants were incubated in 10 mM bromodeoxyuridine (BrdU, Sigma-Aldrich, St Louis, USA) dissolved in system water for 4 h as described with modification [61]. Blood smears were obtained as described previously [62]. Kidney marrow blood cells were fixed by 4% paraformaldehyde, stained with mouse-anti-BrdU (Roche, Basel, Switzerland, cat.#1170376001, 1 : 16) and rabbit-anti-dsRed Abs (Clontech, Mountain View, USA, cat.#632496, 1 : 400), coupled with Alexa Fluor 488 anti-mouse (Abcam, England, cat.#ab150153, 1 : 400) and Alexa 555 anti-rabbit (Abcam, England, cat.#ab150078, 1 : 400) for fluorescent observation. The ratio of *rag2:dsRed* and BrdU co-staining cells in *rag2:dsRed^+^* cells was calculated between *runx1^+/+^* and *runx1^W84X/W84X^* mutants. TUNEL assay was conducted using In-situ Cell Death Detection Kit (Roche, Basel, Switzerland) as described [63], coupled with rabbit-anti-dsRed Abs (Clontech, Mountain View, USA, cat.#632496, 1 : 400) and Alexa 555 anti-rabbit (Abcam, England, cat.#ab150078, 1 : 400). The ratio of *rag2:dsRed* and TUNEL co-staining cells in *rag2:dsRed^+^* cells was calculated between *runx1^+/+^* and *runx1^W84X/W84X^* mutants.

### 4.7. Chromatin immunoprecipitation

Embryos were harvested from *hsp70l:myc-runx1*zebrafish and heat shocked at 39°C. Crosslinked chromatin was immunoprecipitated with anti-Myc antibody (MBL, Japan, cat.#m192-3, 1 : 63) or IgG (negative control, Invitrogen, USA, cat.#10003D) according to the procedure described by Hart *et al.* [64]. The immunoprecipitates were subjected to semi-quantitative PCR. The primers used in this assay are listed in table 1.

### 4.8. Transplantation procedure

Kidney marrow transplantation experiments were carried out as described previously [59,65] with some minor modifications. Adult *runx1^+/+^* and *runx1^W84X/W84X^* mutant hosts were γ-irradiated beforehand with a total dose at 25 Gy and then received transplantation after two days without feed. Kidney marrow blood cells suspension from adult donors *Tg(igm:eGFP);runx1^+/+^* and *Tg(igm:eGFP);runx1^W84X/W84X^* mutants were filtered through a 40 µm strainer. Kidney marrow blood cells were calculated manually by a haematocytometer. Counted kidney marrow blood cells were centrifuged at 800 g for 5 min at 4°C, then resuspended in injection medium (PBS with 5% FBS containing 3U Heparin and 1U DNaseI) and divided into desired volumes for the following transplantation. Approx. 10^6^ kidney marrow blood cells from donors were transplanted into the heart of hosts using a glass capillary needle (Eppendorf, Hamburg, Germany). Transplanted hosts were raised very carefully and fed for the next week. The three groups assigned were *Tg(igm:eGFP);runx1^+/+^* donor to *runx1^+/+^* host, *Tg(igm:eGFP);runx1^+/+^* donor to *runx1^W84X/W84X^* mutants host, *Tg(igm:eGFP);runx1^W84X/W84X^* mutants donor to *runx1^+/+^* host. 4 weeks after transplantation, *igm:eGFP^+^* cells and kidneys of hosts were measured by flow cytometry and the fluorescences were observed.

### 4.9. Imaging analysis

Images of blood smear samples were captured on an Olympus DP 71 microscope (Olympus, Tokyo, Japan) and a Zeiss confocal microscope (ZEISS LSM 510, Germany). Adult zebrafish were observed using a ZEISS microscope (ZEISS Discovery. v. 20, Germany) and photographed by an Olympus MVX10 microscope (Olympus, Tokyo, Japan). All the images were handled by Adobe Photoshop CS5 (Adobe, San Jose).

### 4.10. Statistical analysis

The data were organized by the two-tailed Student's *t*-test or ANOVA, while comparison of survival curves was performed with the log-rank test. And the data were shown as a mean ± s.e. of the mean (s.e.m). Differences were considered significant when the *P* value was less than 0.05. Statistical analyses were done using graphpad Prism v. 6 (GraphPad Software, La Jolla, USA).

## Supplementary Material

2 supplementary figures

## Data accessibility

All relevant data are included in the manuscript and the electronic supplementary material.

## References

[1] Barnett BE, Ciocca ML, Goenka R, Barnett LG, Wu J, Laufer TM, Burkhardt JK, Cancro MP, Reiner SL 2012 Asymmetric B cell division in the germinal center reaction. Science 335, 342–344. (10.1126/science.1213495) 22174128 PMC3282111

[2] Yasuda T et al. 2016 Corrigendum: recurrent DUX4 fusions in B cell acute lymphoblastic leukemia of adolescents and young adults. Nat. Genet. 48, 1591 (10.1038/ng1216-1587a) 27898077

[3] Rodríguez-Cortez VC, del Pino-Molina L, Rodríguez-Ubreva J, Gómez-Cabrero D, Urquiza JM, Tegnér J, Rodríguez-Gallego C, López-Granados E, Ballestar E 2015 Monozygotic twins discordant for common variable immunodeficiency reveal impaired DNA demethylation during naive-to-memory B-cell transition. Nat. Commun. 6, 7335 (10.1038/ncomms8335) 26081581 PMC4557293

[4] Boushaki S et al. 2015 Prevalence of BTK mutations in male Algerian patterns with agammaglobulinemia and severe B cell lymphopenia. Clin. Immunol. 161, 286–290. (10.1016/j.clim.2015.09.011) 26387629

[5] Li J et al. 2015 Association of CLEC16A with human common variable immunodeficiency disorder and role in murine B cells. Nat. Commun. 6, 6804 (10.1038/ncomms7804) 25891430 PMC4444044

[6] Holmes ML, Pridans C, Nutt SL 2008 The regulation of the B-cell gene expression programme by Pax5. Immunol. Cell Biol. 86, 47–53. (10.1038/sj.icb.7100134) 17998914

[7] Manilay JO, Zouali M 2014 Tight relationships between B lymphocytes and the skeletal system. Trends Mol. Med. 20, 405–412. (10.1016/j.molmed.2014.03.003) 24726716

[8] Libermann TA, Pan Z, Akbarali Y, Hetherington CJ, Boltax J, Yergeau DA, Zhang DE 1999 AML1 (CBFalpha2) cooperates with B cell-specific activating protein (BSAP/PAX5) in activation of the B cell-specific BLK gene promoter. J. Biol. Chem. 274, 24 671–24 676. (10.1074/jbc.274.35.24671) 10455134

[9] Kwon K, Hutter C, Sun Q, Bilic I, Cobaleda C, Malin S, Busslinger M 2008 Instructive role of the transcription factor E2A in early B lymphopoiesis and germinal center B cell development. Immunity 28, 751–762. (10.1016/j.immuni.2008.04.014) 18538592

[10] Hu Y et al. 2016 Superenhancer reprogramming drives a B-cell-epithelial transition and high-risk leukemia. Genes Dev. 30, 1971–1990. (10.1101/gad.283762.116) 27664237 PMC5066240

[11] Liu H, Carlsson L, Grundström T 2006 Identification of an N-terminal transactivation domain of Runx1 that separates molecular function from global differentiation function. J. Biol. Chem. 281, 25 659–25 669. (10.1074/jbc.M603249200) 16803898

[12] Link KA, Chou FS, Mulloy JC 2010 Core binding factor at the crossroads: determining the fate of the HSC. J. Cell. Physiol. 222, 50–56. (10.1002/jcp.21950) 19813271 PMC2812028

[13] Goyama S et al. 2013 Transcription factor RUNX1 promotes survival of acute myeloid leukemia cells. J. Clin. Invest. 123, 3876–3888. (10.1172/JCI68557) 23979164 PMC3754260

[14] Kim W, Barron DA, San Martin R, Chan KS, Tran LL, Yang F, Ressler SJ, Rowley DR 2014 RUNX1 is essential for mesenchymal stem cell proliferation and myofibroblast differentiation. Proc. Natl Acad. Sci. USA 111, 16 389–16 394. (10.1073/pnas.1407097111) PMC4246299 25313057

[15] Sood R, Kamikubo Y, Liu P 2017 Role of RUNX1 in hematological malignancies. Blood 129, 2070–2082. (10.1182/blood-2016-10-687830) 28179279 PMC5391618

[16] Okuda T, van Deursen J, Hiebert SW, Grosveld G, Downing JR 1996 AML1, the target of multiple chromosomal translocations in human leukemia, is essential for normal fetal liver hematopoiesis. Cell 84, 321–330. (10.1016/S0092-8674(00)80986-1) 8565077

[17] Swiers G, de Bruijn M, Speck NA 2010 Hematopoietic stem cell emergence in the conceptus and the role of Runx1. Int. J. Dev. Biol. 54, 1151–1163. (10.1387/ijdb.103106gs) 20711992 PMC4512753

[18] Bresciani E, Carrington B, Wincovitch S, Jones M, Gore AV, Weinstein BM, Sood R, Liu PP 2014 CBF*β* and RUNX1 are required at 2 different steps during the development of hematopoietic stem cells in zebrafish. Blood 124, 70–78. (10.1182/blood-2013-10-531988) 24850758 PMC4125354

[19] Antony-Debré I et al. 2012 MYH10 protein expression in platelets as a biomarker of RUNX1 and FLI1 alterations. Blood 120, 2719–2722. (10.1182/blood-2012-04-422352) 22677128

[20] Seo W, Ikawa T, Kawamoto H, Taniuchi I 2012 Runx1-Cbfbeta facilitates early B lymphocyte development by regulating expression of Ebf1. J. Exp. Med. 209, 1255–1262. (10.1084/jem.20112745) 22665574 PMC3405506

[21] Niebuhr B et al. 2013 Runx1 is essential at two stages of early murine B-cell development. Blood 122, 413–423. (10.1182/blood-2013-01-480244) 23704093

[22] Zaliova M et al. 2017 ETV6/RUNX1-like acute lymphoblastic leukemia: a novel B-cell precursor leukemia subtype associated with the CD27/CD44 immunophenotype. Genes Chromosomes Cancer 56, 608–616. (10.1002/gcc.22464) 28395118

[23] Grossmann V, Kern W, Harbich S, Alpermann T, Jeromin S, Schnittger S, Haferlach C, Haferlach T, Kohlmann A 2011 Prognostic relevance of RUNX1 mutations in T-cell acute lymphoblastic leukemia. Haematologica 96, 1874–1877. (10.3324/haematol.2011.043919) 21828118 PMC3232273

[24] Tian Y et al. 2017 The first wave of T lymphopoiesis in zebrafish arises from aorta endothelium independent of hematopoietic stem cells. J. Exp. Med. 214, 3347–3360. (10.1084/jem.20170488) 28931624 PMC5679161

[25] Iwanami N 2014 Zebrafish as a model for understanding the evolution of the vertebrate immune system and human primary immunodeficiency. Exp. Hematol. 42, 697–706. (10.1016/j.exphem.2014.05.001) 24824573

[26] Yan B, Han P, Pan L, Lu W, Xiong J, Zhang M, Zhang W, Li L, Wen Z 2014 IL-1beta and reactive oxygen species differentially regulate neutrophil directional migration and Basal random motility in a zebrafish injury-induced inflammation model. J. Immunol. 192, 5998–6008. (10.4049/jimmunol.1301645) 24835391

[27] Willett CE, Cortes A, Zuasti A, Zapata AG 1999 Early hematopoiesis and developing lymphoid organs in the zebrafish. Dev. Dyn. 214, 323–336. (10.1002/(SICI)1097-0177(199904)214:4%3C323::AID-AJA5%3E3.0.CO;2-3) 10213388

[28] Trede NS, Zapata A, Zon LI 2001 Fishing for lymphoid genes. Trends Immunol. 22, 302–307. (10.1016/S1471-4906(01)01939-1) 11377288

[29] Rijkers GT, Frederix-Wolters EM, van Muiswinkel WB 1980 The immune system of cyprinid fish. Kinetics and temperature dependence of antibody-producing cells in carp (*Cyprinus carpio*). Immunology 41, 91–97. 7000695 PMC1458243

[30] Taniuchi I, Osato M, Egawa T, Sunshine MJ, Bae SC, Komori T, Ito Y, Littman DR 2002 Differential requirements for Runx proteins in CD4 repression and epigenetic silencing during T lymphocyte development. Cell 111, 621–633. (10.1016/S0092-8674(02)01111-X) 12464175

[31] Palmi C et al. 2014 Cytoskeletal regulatory gene expression and migratory properties of B-cell progenitors are affected by the ETV6-RUNX1 rearrangement. Mol. Cancer Res. 12, 1796–1806. (10.1158/1541-7786.MCR-14-0056-T) 25061103

[32] Jin H, Sood R, Xu J, Zhen F, English MA, Liu PP, Wen Z 2009 Definitive hematopoietic stem/progenitor cells manifest distinct differentiation output in the zebrafish VDA and PBI. Development 136, 647–654. (10.1242/dev.029637) 19168679 PMC2646468

[33] Sood R et al. 2010 Development of multilineage adult hematopoiesis in the zebrafish with a runx1 truncation mutation. Blood 115, 2806–2809. (10.1182/blood-2009-08-236729) 20154212 PMC2854427

[34] Langenau DM, Ferrando AA, Traver D, Kutok JL, Hezel JP, Kanki JP, Zon LI, Look AT, Trede NS 2004 In vivo tracking of T cell development, ablation, and engraftment in transgenic zebrafish. Proc. Natl Acad. Sci. USA 101, 7369–7374. (10.1073/pnas.0402248101) 15123839 PMC409925

[35] Page DM et al. 2013 An evolutionarily conserved program of B-cell development and activation in zebrafish. Blood 122, e1–e11. (10.1182/blood-2012-12-471029) 23861249 PMC3750348

[36] Lukin K, Fields S, Lopez D, Cherrier M, Ternyak K, Ramírez J, Feeney AJ, Hagman J 2010 Compound haploinsufficiencies of Ebf1 and Runx1 genes impede B cell lineage progression. Proc. Natl Acad. Sci. USA 107, 7869–7874. (10.1073/pnas.1003525107) 20385820 PMC2867885

[37] Wang Z, Wu Y, Hu Q, Li Y 2015 Differences on the biological function of three Ig isotypes in zebrafish: a gene expression profile. Fish Shellfish Immunol. 44, 283–286. (10.1016/j.fsi.2015.02.030) 25725401

[38] Ungerbäck J, Ahsberg J, Strid T, Somasundaram R, Sigvardsson M 2015 Combined heterozygous loss of Ebf1 and Pax5 allows for T-lineage conversion of B cell progenitors. J. Exp. Med. 212, 1109–1123. (10.1084/jem.20132100) 26056231 PMC4493409

[39] Lin AF, Xiang LX, Wang QL, Dong WR, Gong YF, Shao JZ 2009 The DC-SIGN of zebrafish: insights into the existence of a CD209 homologue in a lower vertebrate and its involvement in adaptive immunity. J. Immunol. 183, 7398–7410. (10.4049/jimmunol.0803955) 19890038

[40] Kuehn HS et al. 2016 Loss of B cells in patients with heterozygous mutations in IKAROS. N. Engl. J. Med. 374, 1032–1043. (10.1056/NEJMoa1512234) 26981933 PMC4836293

[41] Wienholds E, Schulte-Merker S, Walderich B, Plasterk RH 2002 Target-selected inactivation of the zebrafish rag1 gene. Science 297, 99–102. (10.1126/science.1071762) 12098699

[42] Schorpp M, Bialecki M, Diekhoff D, Walderich B, Odenthal J, Maischein HM, Zapata AG, Boehm T 2006 Conserved functions of Ikaros in vertebrate lymphocyte development: genetic evidence for distinct larval and adult phases of T cell development and two lineages of B cells in zebrafish. J. Immunol. 177, 2463–2476. (10.4049/jimmunol.177.4.2463) 16888008

[43] Petrie-Hanson L, Hohn C, Hanson L 2009 Characterization of rag1 mutant zebrafish leukocytes. Bmc Immunol. 10, 8 (10.1186/1471-2172-10-8) 19192305 PMC2645361

[44] Sun J et al. 2013 Suppression of Pu.1 function results in expanded myelopoiesis in zebrafish. Leukemia 27, 1913–1917. (10.1038/leu.2013.67) 23455395

[45] Györy I, Boller S, Nechanitzky R, Mandel E, Pott S, Liu E, Grosschedl R 2012 Transcription factor Ebf1 regulates differentiation stage-specific signaling, proliferation, and survival of B cells. Genes Dev. 26, 668–682. (10.1101/gad.187328.112) 22431510 PMC3323878

[46] Mandel EM, Grosschedl R 2010 Transcription control of early B cell differentiation. Curr. Opin. Immunol. 22, 161–167. (10.1016/j.coi.2010.01.010) 20144854

[47] Parker L, Stainier DY 1999 Cell-autonomous and non-autonomous requirements for the zebrafish gene cloche in hematopoiesis. Development 126, 2643–2651. 10331976 10.1242/dev.126.12.2643

[48] Wang L et al. 2013 Fev regulates hematopoietic stem cell development via ERK signaling. Blood 122, 367–375. (10.1182/blood-2012-10-462655) 23591790

[49] Hess I, Boehm T 2016 Stable multilineage xenogeneic replacement of definitive hematopoiesis in adult zebrafish. Sci. Rep. 6, 19634 (10.1038/srep19634) 26777855 PMC4726038

[50] Chimienti G, Alaibac M, Marzullo F, Carbone A, Pepe G 2000 The expression pattern of the AML1 gene in non-Hodgkin's B-cell lymphomas and normal B lymphocytes. Blood Cells Mol. Dis. 26, 186–192. (10.1006/bcmd.2000.0295) 10950938

[51] Bäsecke J, Feuring-Buske M, Brittinger G, Schaefer UW, Hiddemann W, Griesinger F 2002 Transcription of AML1 in hematopoietic subfractions of normal adults. Ann. Hematol. 81, 254–257. (10.1007/s00277-002-0453-8) 12029534

[52] Lorsbach RB, Moore J, Ang SO, Sun W, Lenny N, Downing JR 2004 Role of RUNX1 in adult hematopoiesis: analysis of RUNX1-IRES-GFP knock-in mice reveals differential lineage expression. Blood 103, 2522–2529. (10.1182/blood-2003-07-2439) 14630789

[53] Schebesta A, McManus S, Salvagiotto G, Delogu A, Busslinger GA, Busslinger M 2007 Transcription factor Pax5 activates the chromatin of key genes involved in B cell signaling, adhesion, migration, and immune function. Immunity 27, 49–63. (10.1016/j.immuni.2007.05.019) 17658281

[54] Lam QL, Lo CK, Zheng BJ, Ko KH, Osmond DG, Wu GE, Rottapel R, Lu L 2007 Impaired V(D)J recombination and increased apoptosis among B cell precursors in the bone marrow of c-Abl-deficient mice. Int. Immunol. 19, 267–276. (10.1093/intimm/dxl143) 17229817

[55] Westerfield M 1995 The zebrafish book: a guide for the laboratory use of zebrafish *Danio (Brachydanio) rerio*. Oregon, OR: USA: Oregon University Press.

[56] Willett CE, Cherry JJ, Steiner LA 1997 Characterization and expression of the recombination activating genes (rag1 and rag2) of zebrafish. Immunogenetics 45, 394–404. (10.1007/s002510050221) 9089097

[57] Jin H, Li L, Xu J, Zhen F, Zhu L, Liu PP, Zhang M, Zhang W, Wen Z 2012 Runx1 regulates embryonic myeloid fate choice in zebrafish through a negative feedback loop inhibiting Pu.1 expression. Blood 119, 5239–5249. (10.1182/blood-2011-12-398362) 22493295 PMC3369614

[58] Stachura DL, Traver D 2016 Cellular dissection of zebrafish hematopoiesis. Methods Cell Biol. 133, 11–53. (10.1016/bs.mcb.2016.03.022) 27263407

[59] Traver D, Paw BH, Poss KD, Penberthy WT, Lin S, Zon LI 2003 Transplantation and in vivo imaging of multilineage engraftment in zebrafish bloodless mutants. Nat. Immunol. 4, 1238–1246. (10.1038/ni1007) 14608381

[60] Weir H, Chen PL, Deiss TC, Jacobs N, Nabity MB, Young M, Criscitiello MF 2015 DNP-KLH yields changes in leukocyte populations and immunoglobulin isotype use with different immunization routes in zebrafish. Front. Immunol. 6, 606 (10.3389/fimmu.2015.00606) 26648935 PMC4664633

[61] Wallace KN, Akhter S, Smith EM, Lorent K, Pack M 2005 Intestinal growth and differentiation in zebrafish. Mech. Dev. 122, 157–173. (10.1016/j.mod.2004.10.009) 15652704

[62] Balla KM, Lugo-Villarino G, Spitsbergen JM, Stachura DL, Hu Y, Banuelos K, Romo-Fewell O, Aroian RV, Traver D 2010 Eosinophils in the zebrafish: prospective isolation, characterization, and eosinophilia induction by helminth determinants. Blood 116, 3944–3954. (10.1182/blood-2010-03-267419) 20713961 PMC2981543

[63] Du L, Xu J, Li X, Ma N, Liu Y, Peng J, Osato M, Zhang W, Wen Z 2011 Rumba and Haus3 are essential factors for the maintenance of hematopoietic stem/progenitor cells during zebrafish hematopoiesis. Development 138, 619–629. (10.1242/dev.054536) 21228005

[64] Hart DO, Raha T, Lawson ND, Green MR 2007 Initiation of zebrafish hematopoiesis by the TATA-box-binding protein-related factor, Trf3. Nature 450, 1082–1085. (10.1038/nature06349) 18046332 PMC2150749

[65] Liu W et al. 2017 c-myb hyperactivity leads to myeloid and lymphoid malignancies in zebrafish. Leukemia 31, 222–233. (10.1038/leu.2016.170) 27457538 PMC5972522

