# Supplementary material for: Loss of runx1 function results in B cell immunodeficiency but not T cell in adult zebrafish
Source: Open Biol. 2018 Jul 25;8(7):180043. doi: 10.1098/rsob.180043 (PMC6070721; doi:10.1098/rsob.180043)
Supplement: 2 supplementary figures [file rsob180043supp1.pdf]

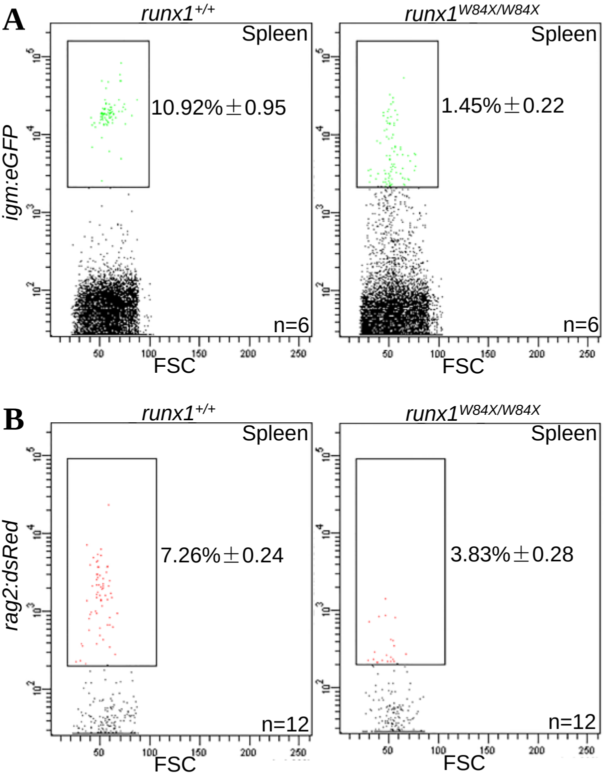


**Supplementary Figure 1.** Decreased B cells in spleen of *runx1*$^{W84X/W84X}$ mutants. **A**-**B**: Percentage analysis of B cell makers *igm* (**A**) and *rag2* (**B**) in spleen of WT and *runx1*$^{W84X/W84X}$ mutants by FACS. Black boxes outlined the captured positive lymphocytes with fluorescence in spleen. The percentage data represented the mean±s.e.m.

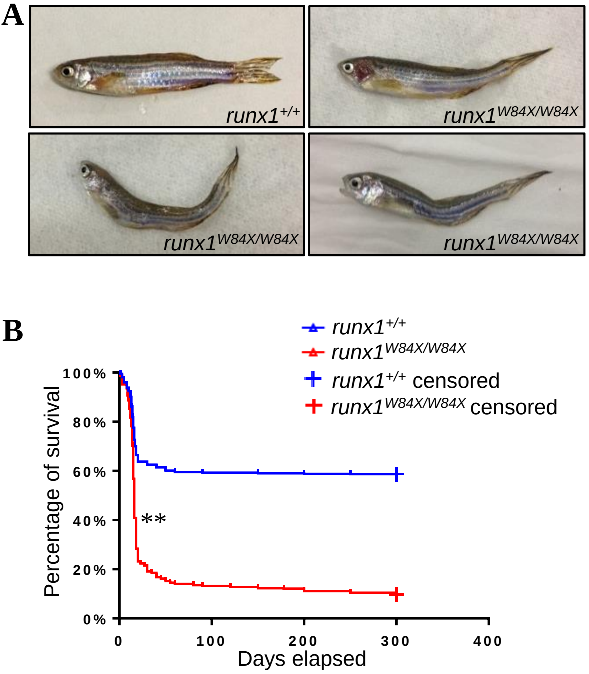


**Supplementary Figure 2.** Fragile growth in *runx1*$^{W84X/W84X}$ mutants. **A:** Pictures showing morphology of WT and hypoplasia of dying *runx1*$^{W84X/W84X}$ mutants. **B:** Survival curve of WT and *runx1*$^{W84X/W84X}$ mutants. log-rank test, ***P<0.01*.
